# Supplementary material for: BRN2 is a non-canonical melanoma tumor-suppressor
Source: Nat Commun. 2021 Jun 17;12:3707. doi: 10.1038/s41467-021-23973-5 (PMC8211827; doi:10.1038/s41467-021-23973-5)
Supplement: Supplementary file 1 — Supplementary Information [file 41467_2021_23973_MOESM1_ESM.pdf]

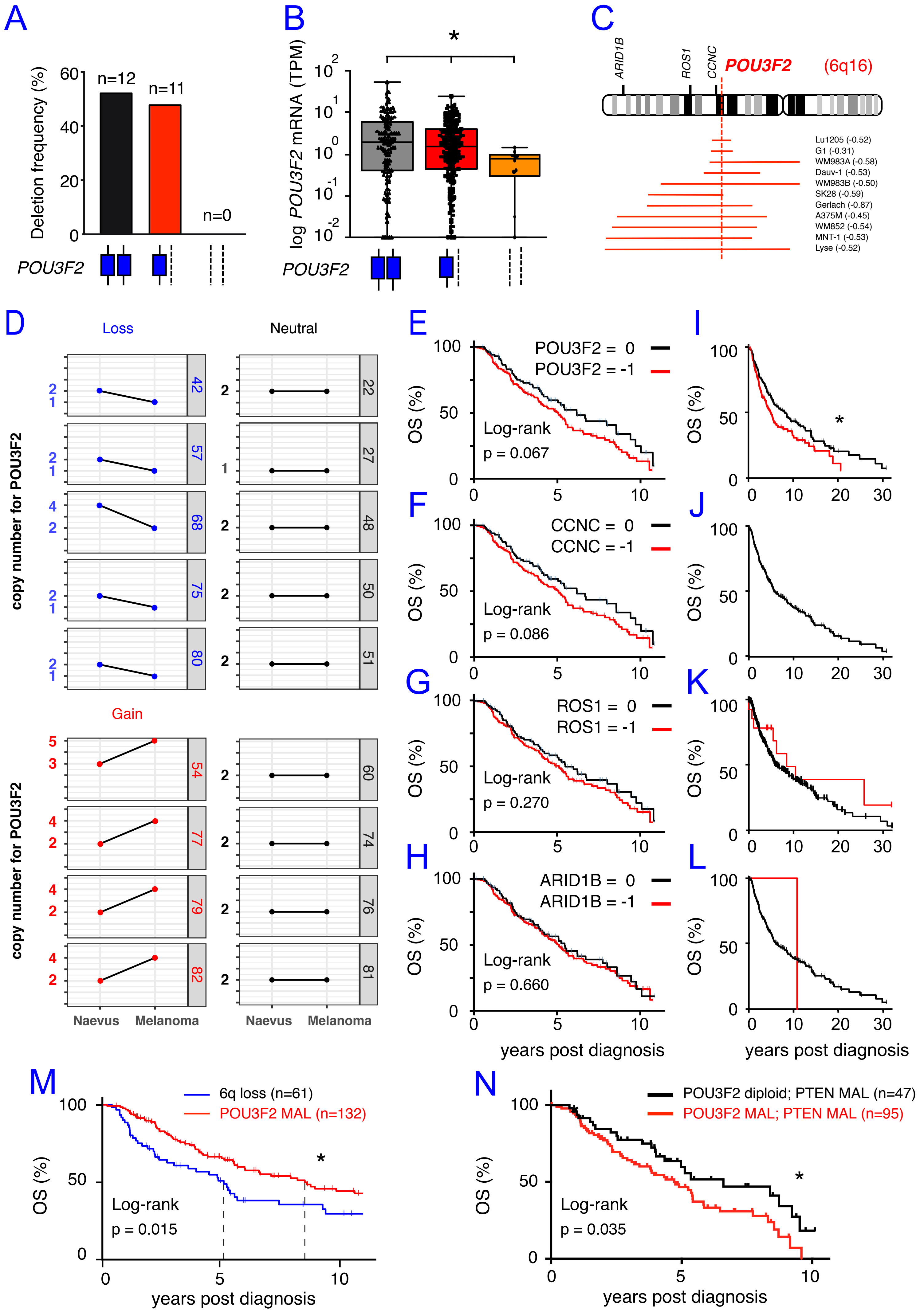

Supplementary Figure 1

Hamm et al.

Supplementary Fig. 1.

(A) *POU3F2* is frequently affected by large deletions in 23 human melanoma cell lines.

(B) *POU3F2* loss is associated with lower mRNA levels in human melanoma.

Box and whisker plot (min to max – median) of correlation between BRN2 mRNA levels and *POU3F2* CNAs in human SKCM metastases. BRN2 mRNA was normalized to transcripts per million (TPM). Statistical analysis were performed using ANOVA (A), *$p < 0.05$.

(C) Extent of segmental deletion affecting BRN2 locus on Chr.6q16 (dashed red line) in melanoma cell lines. The name of cell line and log2 ratio from comparative genomic hybridization is indicated.

(**D**) POU3F2 CNA of matched nevus-melanoma pairs. Melanoma corresponds to primary melanoma except for sample 57, a melanoma in situ. Absolute copy numbers are given on the left and sample numbers are indicated on the right of each scheme.

(**E-L**). Overall survival according to copy number status and gene expression of a set of 6q melanoma-related genes. (**E-H**). Kaplan-Meier curves comparing 10-year overall survival (OS) of SKCM patients whose melanoma expressed a set of melanoma-related genes on 6q chromosome above 1 transcript per million (TPM) (black line) with those whose melanoma did not express the corresponding gene below 1 TPM (red). TCGA RNA-Seq data set was analyzed (n = 309). Gene expression *vs.* no gene expression: log-rank test. (**I-L**). Kaplan-Meier curves comparing 30-year overall survival of SKCM patients for the genes defined above. The TCGA RNA-Seq data set was analyzed (n = 473). (**E,I)** corresponds to POU3F2, (**F,J)** CCNC, (**G,K**) ROS1, and (**H,L**) ARID1B. Note that there was no case in which CCNC gene-expression was below 1 TPM threshold. Analysis was performed using clinical follow-up data last updated by TCGA on August 8, 2019.

(**M**) Kaplan-Meier curves comparing 10-year overall survival of SKCM patients with a 6q loss (blue) or *POU3F2* mono-allelic loss (MAL).

(**N**) *POU3F2* loss is associated with reduced overall survival in PTEN mono-allelic loss (MAL) patients. Ten-year overall survival of melanoma patients with mono-allelic deletion of the *PTEN* gene according to mono-allelic deletion / absence of deletion (diploid) of the *BRN2* gene. Two patients were bi-allelic.

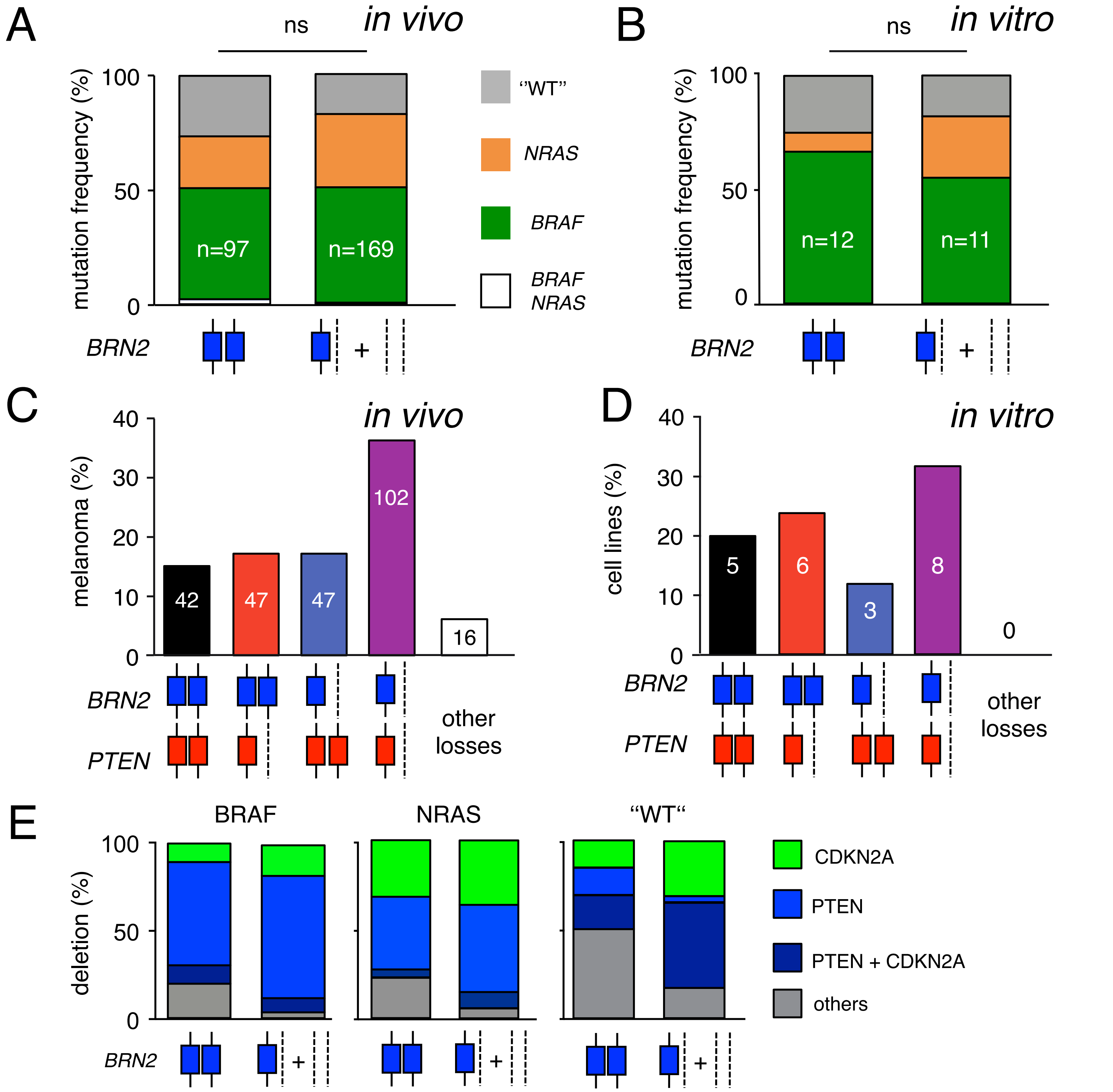

Supplementary Fig. 2. BRN2 is frequently lost in human SKCM and melanoma cell lines independently of NRAS and BRAF driver mutations or CNAs of CDKN2A, and PTEN. (A) Bar graph showing the correlation between the mutation frequency of BRAF (green), NRAS (orange), BRAF + NRAS (white), and "WT" (i.e. non-BRAF/NRAS [grey]) with BRN2 CNAs in human SKCM. CNAs were estimated from GISTIC values. The BRN2 loss group was defined as all samples with mono- or bi-allelic BRN2 loss. BRN2 CNA amplifications (GISTIC > +1) are not shown (n = 21). TCGA sequencing data set (n = 267). Note that from the TCGA_SKCM, only one POU3F2 missense mutation was reported outside of the functional domains. BRN2 allele is depicted as a blue rectangle. (B) Bar graph showing the correlation of mutation frequency between BRAF (green), NRAS (orange), or "WT" (grey) with BRN2 CNAs in human melanoma cell lines (n = 23). (C) Bar graph showing the frequency and co-occurrence of BRN2 and PTEN diploidy and mono-allelic loss in human SKCM. CNAs of BRN2 and PTEN are indicated in the pictograms under the graph. TCGA CNA data-set (n = 362). Note there are amplification of PTEN and/or BRN2 in 108 cases. (D) Bar graph showing the frequency and co-occurrence of BRN2 and PTEN diploidy and mono-allelic loss in human melanoma cell lines (n = 23). CNAs of BRN2 and PTEN are indicated in the pictograms under the graph. One cell line showed a mono-allelic gain of PTEN. (E) Bar graph showing the correlation between BRN2 loss and CNAs of CDKN2A, PTEN, and β-catenin in human SKCM samples harboring either BRAF or NRAS mutations or no BRAF/NRAS mutation. CNAs were estimated from GISTIC values. CNAs of BRN2 are indicated in the pictograms under the graph. TCGA CNA data-set (n = 387). BRAF mutants included P318S/L, P367S, G466E, S467L, L485F, G469A/R, N581T, D594N, L597Q, V600K/E/M/G, K601E, E695K, and H725Y. NRAS mutants included G12A, G13D, Q61H/R/K/L, and E62K. PTEN and CDKN2A mutants included full or partial deletions. Statistical analysis was performed using the Chi-square test. ns = non-significant.

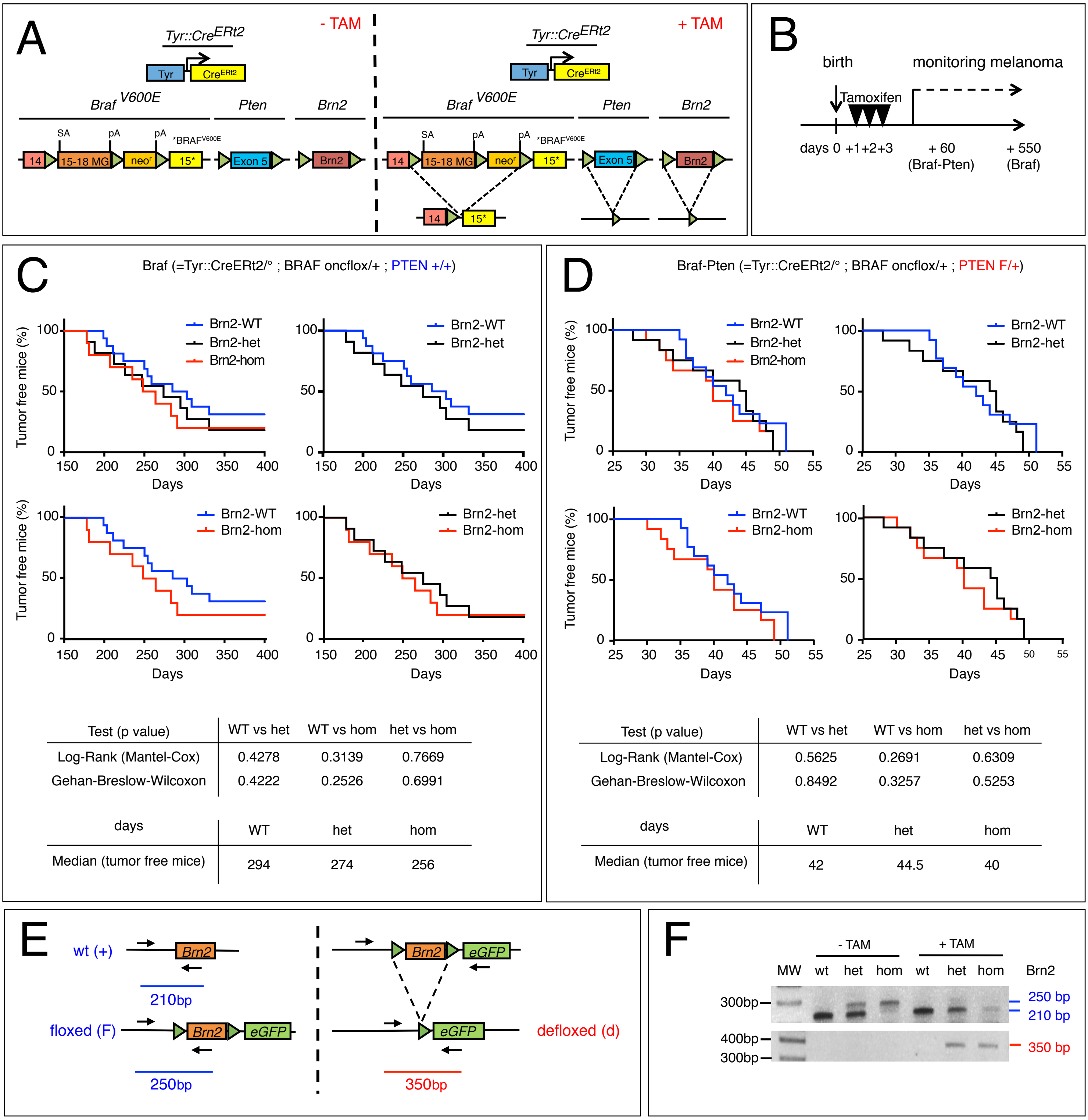

Supplementary Fig. 3. Genetic design, appearance of the first melanoma and characterization of BRN2 locus knock-out mice.
(A) Scheme of the tamoxifen (TAM)-inducible conditional knock-out strategy for Braf, Pten, and Brn2 in the melanocyte lineage. (B) Scheme of TAM application (20 µL/day/mouse [20 µg/mL in DMSO]) for defloxing, leading to the induction of gene knock-out, and mouse follow-up. (C,D) Appearance of the Braf-Brn2 (C) and Braf-Pten-Brn2 (D) tumors. Braf-Brn2-WT (n=16), Braf-Brn2-het (n=11), Braf-Brn2-hom (n=10), Braf-Pten-Brn2-WT (n=13), Braf-Pten-Brn2-het (n=12) and Braf-Pten-Brn2-hom (n=12) mice. The Kaplan-Meier curves between the different Braf (or Braf-Pten) genotypes are not significantly different using the Mantel-Cox test. (E) Scheme of PCR for the detection of wild-type (wt, +), floxed (F), and defloxed (d) Brn2 alleles in knock-out mice. The arrows indicate the location of the primers and the bars the amplicon length. (F) Representative PCR result for allele identification and defloxing of Brn2. Non-TAM-induced (-TAM) tissue from tails or tissue from TAM-induced tumors (+TAM) from Braf-Pten-WT and Braf-Pten-Brn2 mice.

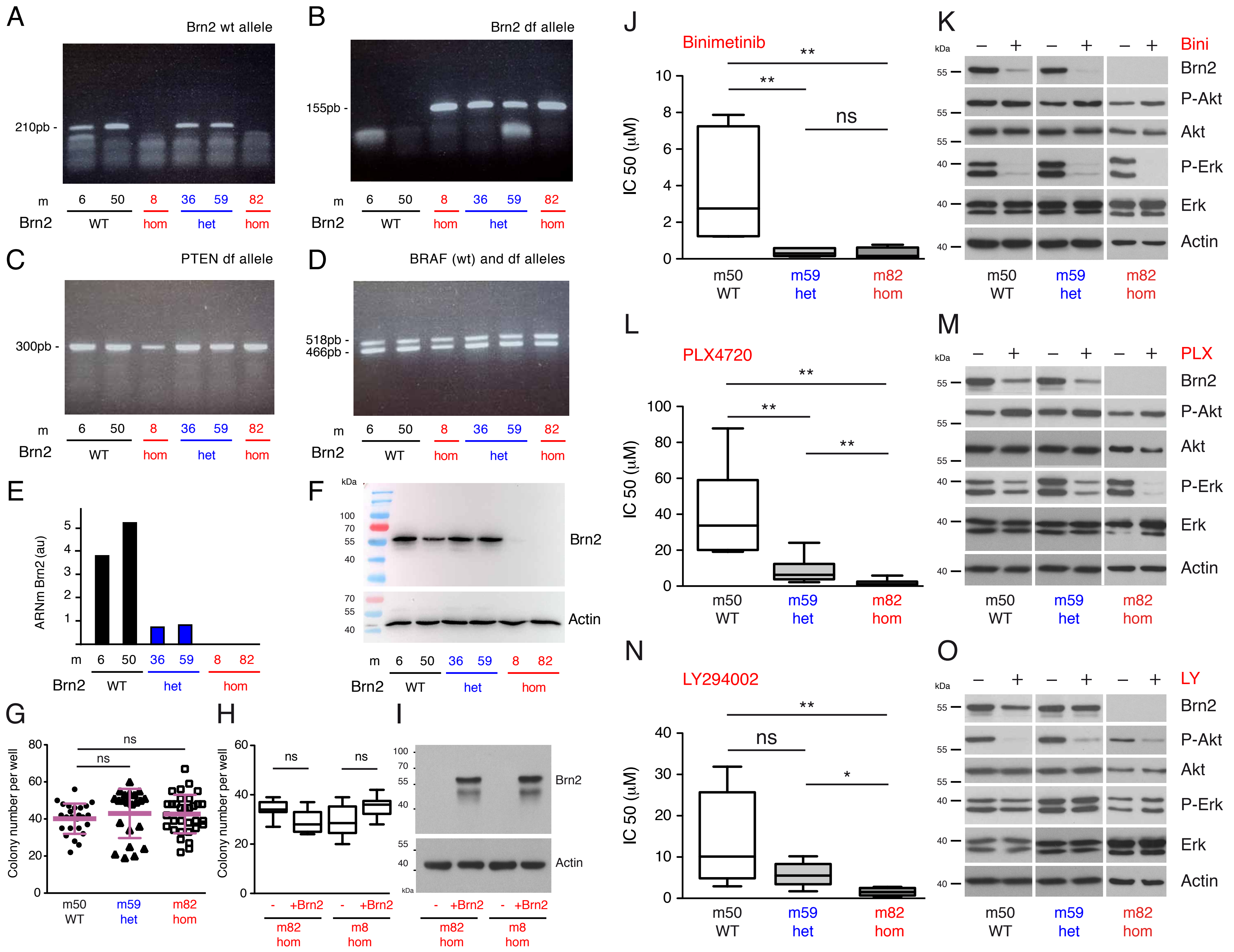

Supplementary Figure 4

Hamm et al

Supplementary Fig. 4. WT mouse melanoma cell lines are more resistant to BRAF, MEK and PI3K inhibitors than het/hom mouse melanoma cell lines.

(A-F) Molecular characterization of established Braf-Pten-Brn2 mouse melanoma cell lines. (**A-D**) The presence of Brn2 WT (**A**) and defloxed alleles (**B**), defloxed allele of PTEN (**C**), and Braf WT and defloxed alleles (**D**) were determined in mentioned cell lines by amplifying the appropriated DNA fragment. Note absence of Brn2 WT allele in two m8/m82 hom cells, and absence of defloxed allele in two m6/m50 WT cells. (**E**) Relative amounts of Brn2 mRNA in mentioned cell lines. (**F**) Western blot analysis using the antibodies mentioned on the left of the panels. Raw data are presented in Supplementary Fig. 9.

(G-I) Level of Brn2 does not affect colony formation in vitro. (**G**) Colony numbers evaluated for different cell lines. Each point represents an independent experiment. n= 25, 22, and 32 for m50, m59 and m82, respectively. Data are presented as mean values +/- SD. ns = non-significant (two-tailed unpaired t-test). (**H**) Colony numbers evaluated for m8/m82 in which BRN2 was re-expressed. Results are presented as Box and Whiskers for seven to eleven independent experiments. ns = non-significant (Mann Whitney test). (**I**) Western blot analysis. (**J,L,N**) IC50 were determined for each pharmacological agent using the colony numbers in mock condition (-) as top response. Cell lines were treated with Binimetinib (**J**) from 10^{-3} to 100 μM, with PLX4720 (**L**) from 10^{-3} to 100 μM, and with LY294002 (**N**) from 5.10^{-3} to 50 μM. n = 4 to 11. Box and Whiskers (min to max - median). ns = non-significant, $^{*}p < 0.05$ and $^{**}p < 0.01$ (Mann Whitney test). (**K,M,O**) Western blot of cell lines incubated for 24 hours with each pharmacological agent. (**K**) 10 μM of Binimetinib, (**M**) 10 μM of PLX4720, and (**O**) 50 μM of LY294002. Raw data are presented in Supplementary Fig. 9. Cells in 2D culture are growing in the presence of 10%FCS and selected for efficient proliferation *in vitro*, which is not the case *in vivo*.

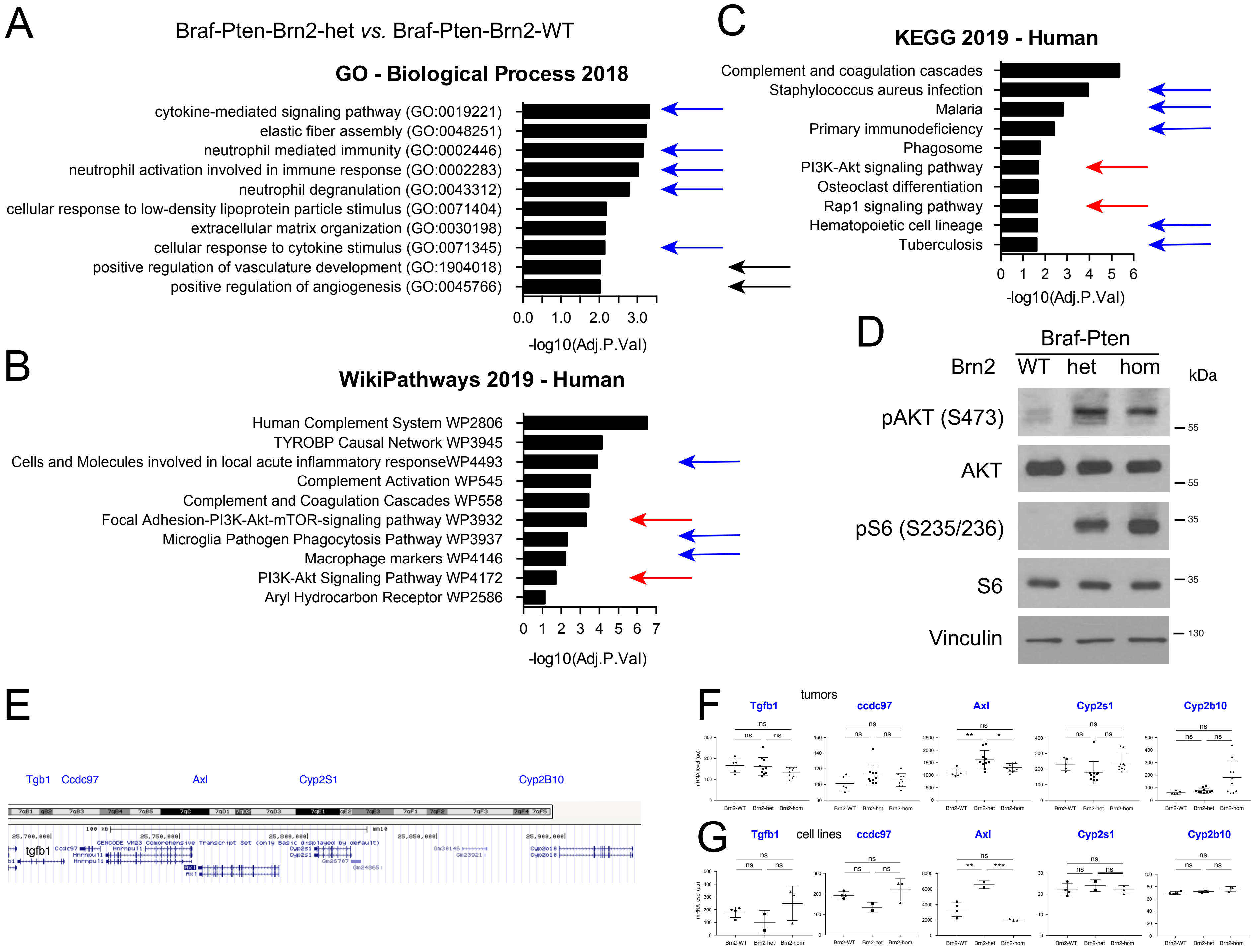

Supplementary Figure 5

Supplementary Fig. 5. An intermediate level of Brn2 (Brn2-het) in a Braf-Pten melanoma induces the immune system, activates PI3K-Akt pathway, and induces AXL.

(A-C) Ontology and pathways enrichment analysis of the 296 genes found overexpressed in the Braf-Pten-Brn2-het tumors compared to the Braf-Pten-Brn2-WT. The blue horizontal arrows marked inflammatory gene sets, the black horizontal arrows marked gene sets involved in angiogenesis and the red horizontal arrows marked gene sets implicated in cell proliferation:

(A) Top 10 biological process from Gene Ontology (GO – Biological process 2018) enriched in the Braf-Pten-Brn2-het signature in tumors. Top 10 enriched pathways from **(B)** WikiPathways (WikiPathways 2019 – Human) and **(C)** KEGG (KEGG 2019 – Human) in the Braf-Pten-Brn2-het signature in tumors.

(D) Western blot showing the activation of AKT, marked by the phosphorylation of the S473 of AKT and the double phosphorylation S235/S236 of S6 – a well-recognized AKT substrate in the Braf-Pten-Brn2-het tumors compared to the Braf-Pten-Brn2-WT. Vinculin is used here as a load control. One representative example is presented, Raw data are presented in Supplementary Fig. 9.

(E) Localization of *Axl* and its two closest upstream genes *Cyp2s1* & *Cyp2b10* and downstream genes *Ccdc97* & *Tgb1* on mouse chromosome 7.

(**F,G**) Expression level of *Axl* and its two closest upstream genes *Cyp2s1* & *Cyp2b10* and its two closest downstream genes *Ccdc97* & *Tgb1* in five, ten and ten WT, het and hom tumors **(F)** and in four, two and three WT, het and hom cell lines, respectively **(G)** according to their Brn2 genetic status highlighting that Axl is finely regulated**.** Statistical analysis was performed using the two-tailed unpaired t-test for F and an Anova test for G. Data are presented as mean values +/- SD for F and G.

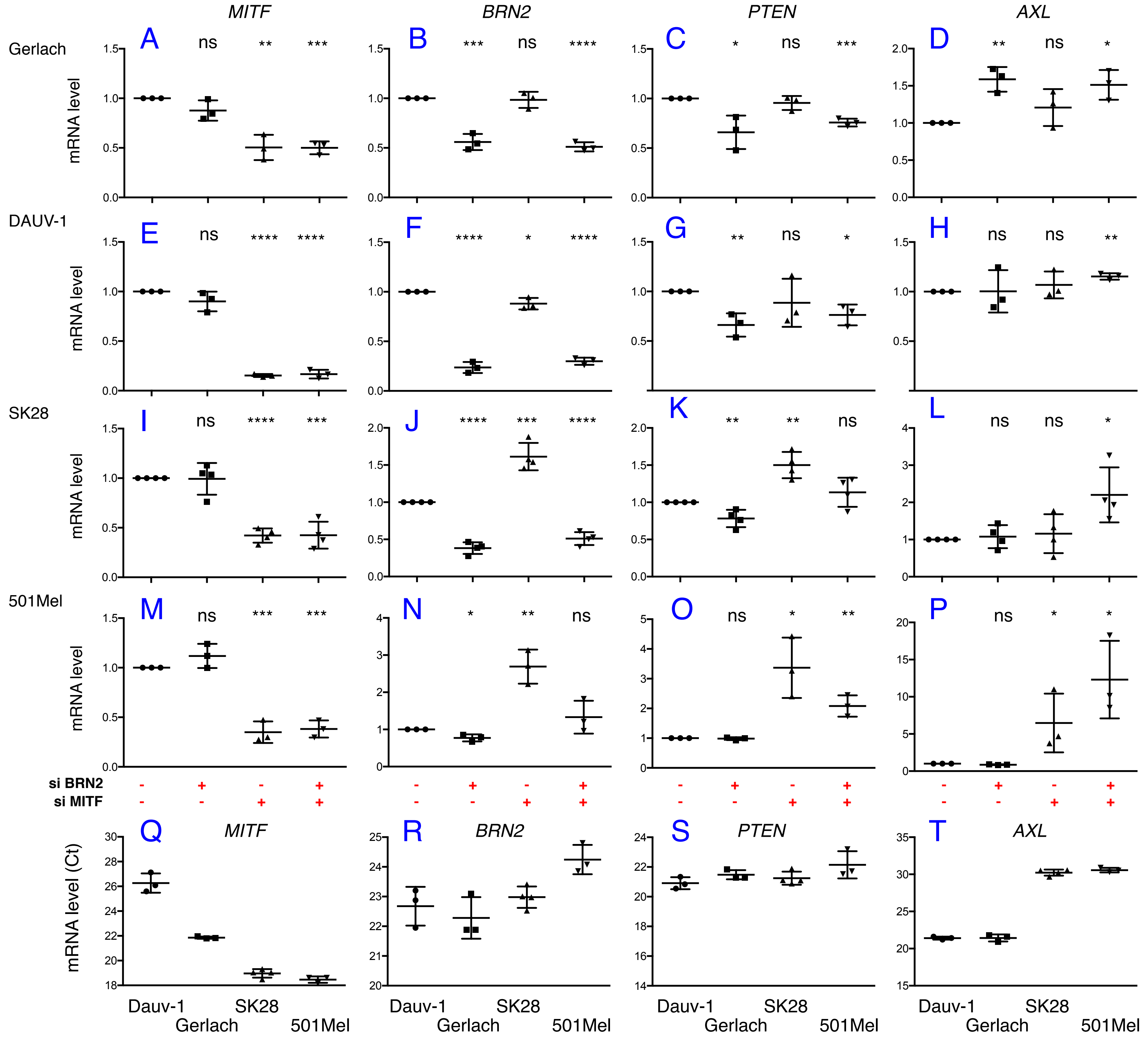

Supplementary Figure 6

Hamm et al

Supplementary Fig. 6. BRN2 and MITF knock down affect the levels of PTEN and AXL mRNA in different human melanoma cell lines.

(**A-P**) The relative amounts of *MITF* (**A,E,I,M)**, *BRN2* (**B,F,J,N)**, *PTEN* (**C,G,K,O),** and *AXL* **(D,H,L,P)** mRNA were determined by RT-qPCR from four human melanoma cell lines (Gerlach [**A-D**], Dauv-1 [**E-H**], SK28 [**I-L**], and 501Mel [**M-P**]) after siRNA-mediated knockdown of BRN2 (pale grey), MITF (dark grey) or both BRN2 and MITF (black), and are given in arbitrary unit. All values were normalized to TBP.

(**Q-T**) Histograms (white) represent the Ct (=Cycle threshold) values of *MITF* (**Q**), *BRN2* (**R**), *PTEN* (**S**), and *AXL* (**T**) obtained by RT-qPCR from si-Scr transfected cells. The analysis was performed on three independent transfections with technical replicates.

Statistical analysis was performed using two-tailed unpaired t-test. Data are presented as mean values +/- SD. ns = non-significant, $*p < 0.05$, $**p < 0.01$ and $***p < 0.001$.

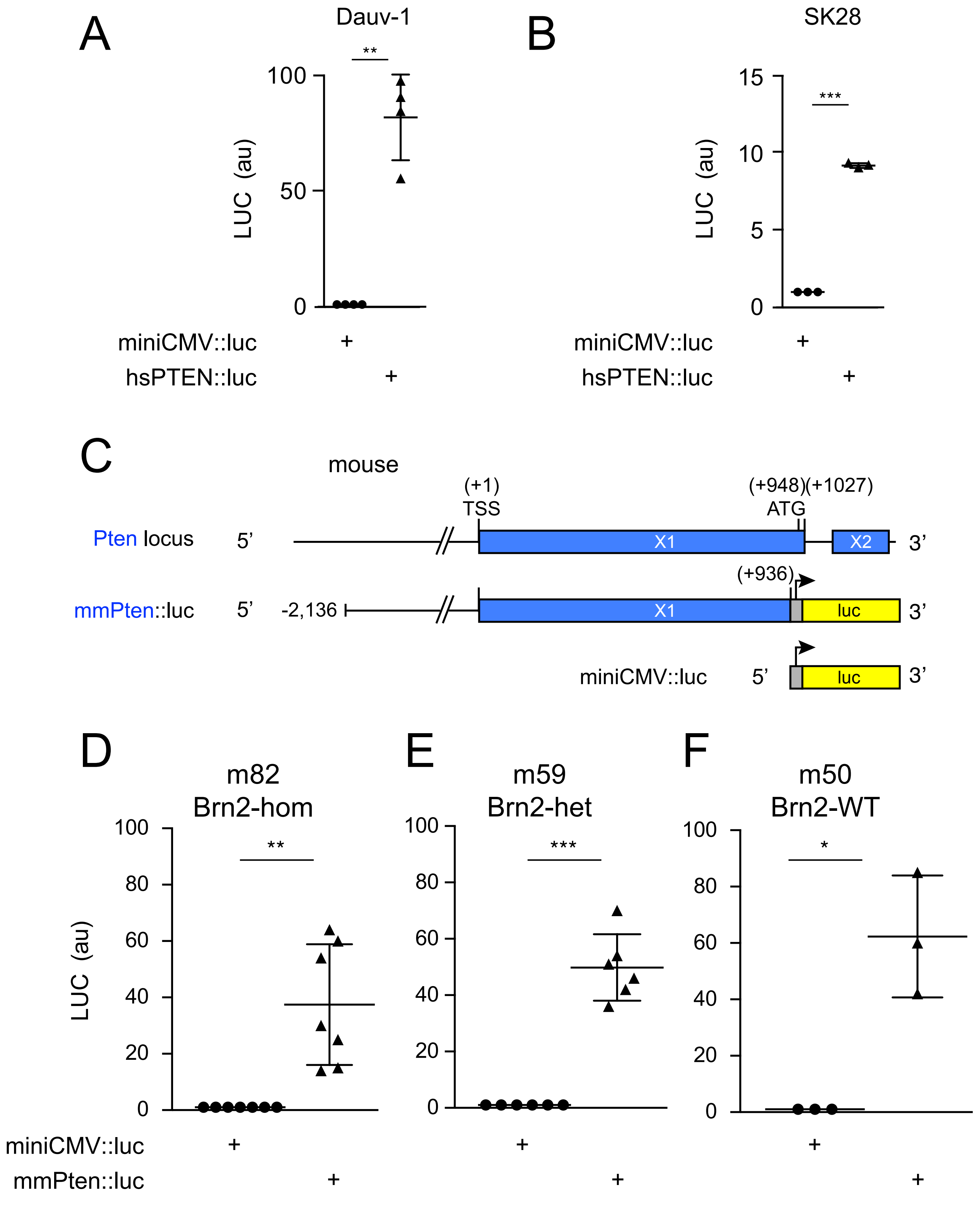

Supplementary Fig. 7. Brn2 activates the human and mouse Pten promoter.
(A,B) Human PTEN promoter and basal mini-CMV promoter activity were evaluated in Dauv-1 (A), SK28 (B) human melanoma cell lines. The experiments were performed independently four and three times evaluating the level of luciferase (LUC), respectively. au means arbitrary unit.(C) Representation of the luciferase reporter construct driven either by the mouse Pten promoter or basal mini-CMV promoter. TSS = transcription start site. Exons (X) 1 and 2 are shown as horizontal rectangles. The translation start site (ATG) and the end of exon 1 are indicated. All numbering is relative to the TSS (+1). (D-F) Mouse Pten promoter and basal mini-CMV promoter luciferase activities were evaluated in mouse m82 [Brn2-hom] (D), m59 [Brn2-het] (E), and m50 [Brn2-WT] (F). The experiments were performed independently seven, six and three times, respectively. Statistical analysis was performed using the two-tailed paired t-test for A, B, D-F. Data are presented as mean values +/- SD. *$p<0.05$, **$p < 0.01$, and ***$p < 0.001$.

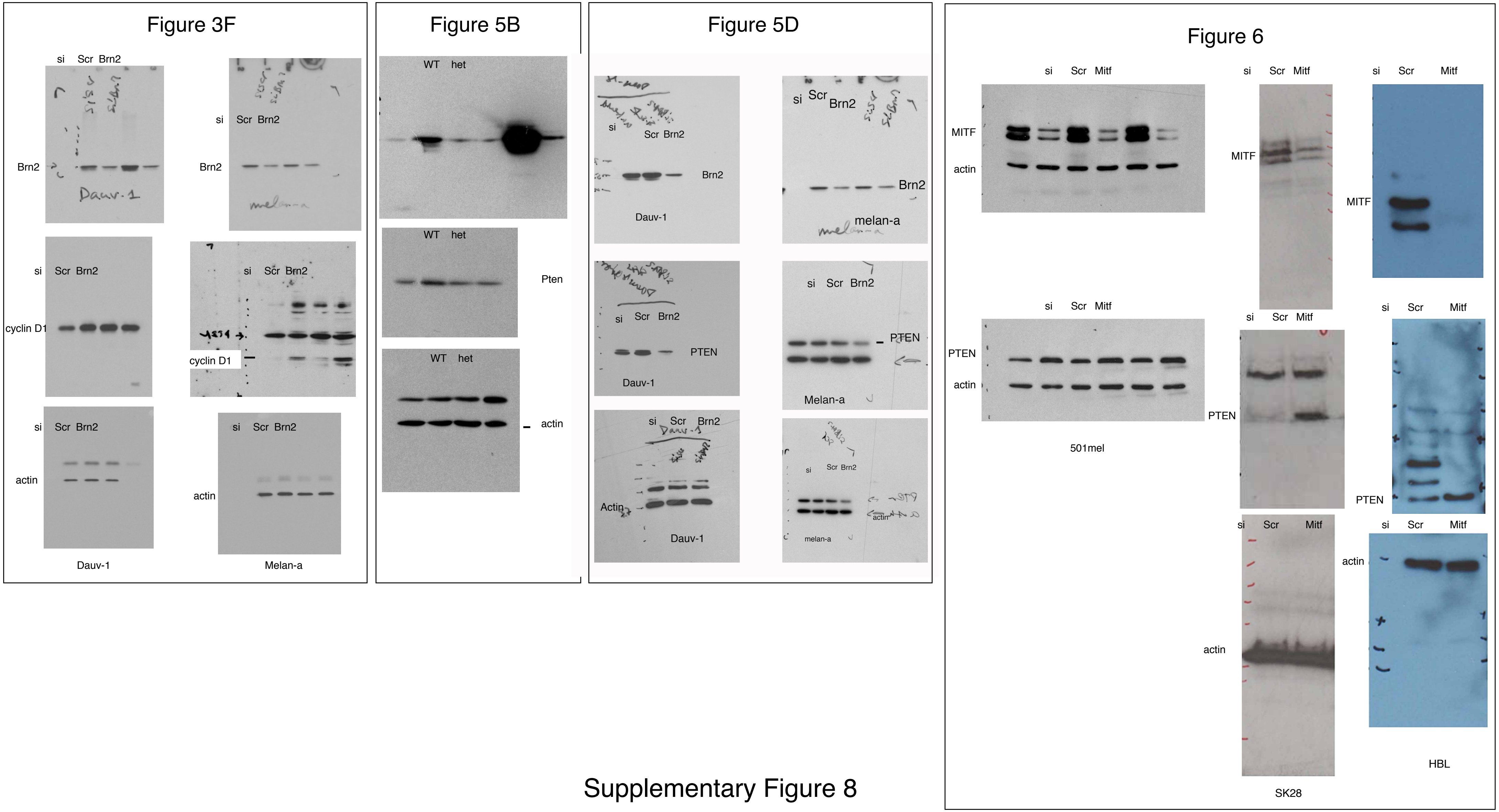

Supplementary Figure 8

Supplementary Figure 9

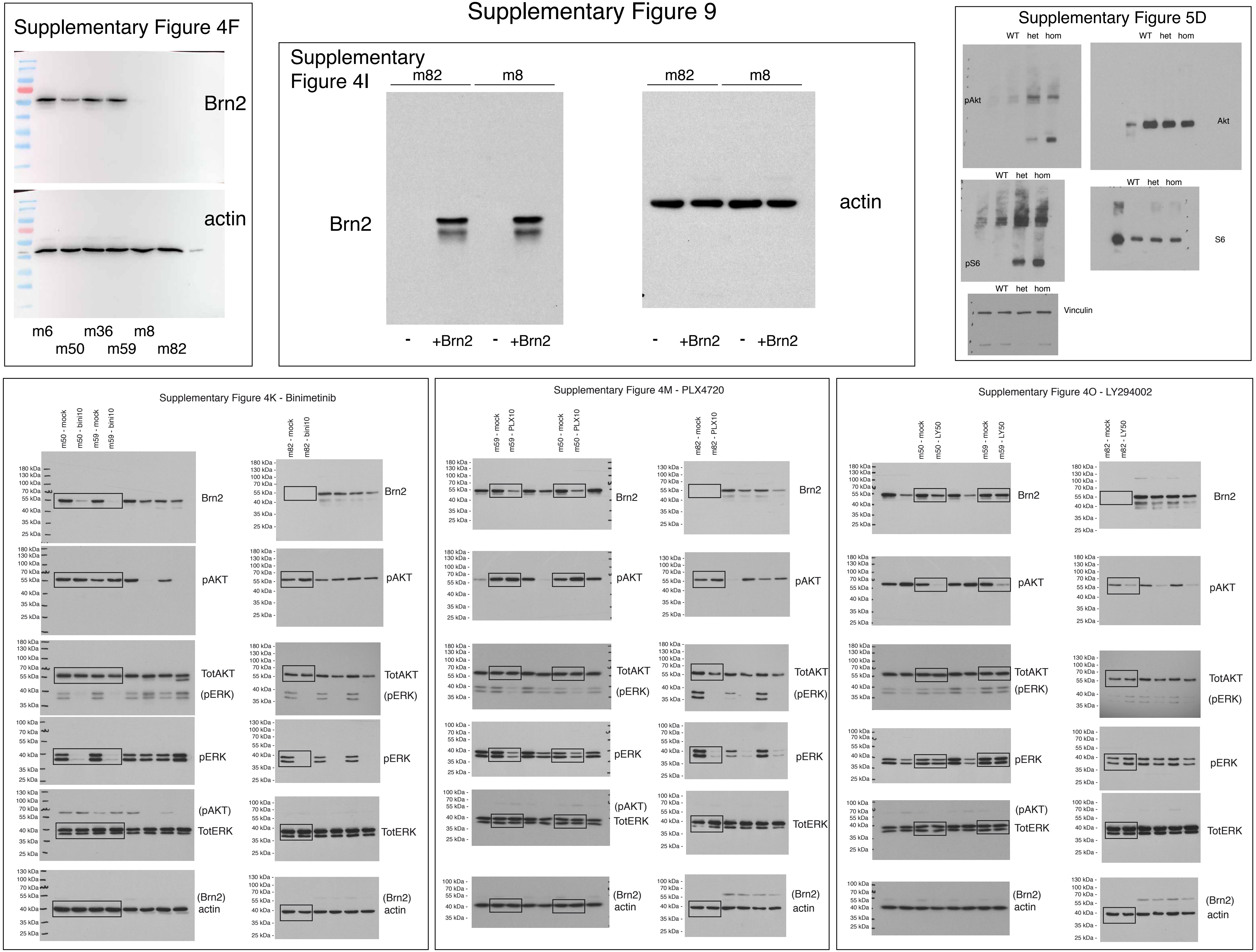

Supplementary Table 1.
BRAF and NRAS genetic status – BRN2 and PTEN protein expression

	genetic status		protein expression	
	**BRAF**	**NRAS**	**BRN2**	**PTEN**
**501mel**	V600E	wt	+	+
**Dauv-1**	V600E	wt	+	+
**Gerlach**	wt	Q61K	+	+
**SK28**	V600E	wt	+	+*
**Melan-a**	wt	wt	+	+

* PTEN is mutated T167A

Supplementary Table 2. Growth of melanoma cell lines in C57BL/6 mice

	Tyr::CreErT2/° ; BrafV600E F/+ ; Pten F/+ ;					
Genotype of the injected cell line	Brn2 +/+	Brn2 +/+	Brn2 F/+	Brn2 F/+	Brn2 F/F	Brn2 F/F
Name of the cell line	m6	m50	m36	m59	m8	m82
Number of tumors / injected mice	4/4	4/4	4/4	4/4	4/4	4/4

Supplementary Table 3. PCR program

Name of PCR program	Temperature	Time (min:sec)	nb of cycles
**ERT2**	94°C	04:00	1 x
	94°C	00:30	
	57°C	00:30	30 x
	72°C	01:00	
	72°C	10:00	1 x
**Classic 58**	94°C	05:00	1 x
	94°C	01:00	
	58°C	01:00	3 x
	72°C	01:00	
	94°C	00:45	
	58°C	00:30	30 x
	72°C	00:30	
	72°C	10:00	1 x
**BRN2**	94°C	03:00	1 x
	94°C	01:00	
	60°C	01:00	35 x
	72°C	01:00	
	72°C	10:00	
**Standard RT-qPCR**	50°C	02:00	
	95°C	10:00	1 x
	95°C	00:15	40 x
	60°C	01:00	
	95°C	00:15	
	60°C	01:00	1 x
	95°C	00:15	

Supplementary Table 4. Oligonucleotides

primer list	name	sequence (5' -> 3')	sense	species	target	genomic location	purpose	primer combination	PCR program*	amplificon size (bp)
RT-Primers	LL1735	TATGGCAACGTGTTCTCGCA	forward	human	BRN2	BRN2 Exon	Detection of mRNA level by RT-qPCR	LL1735 + LL1736	Standard RT-qPCR	109
RT-Primers	LL1736	CCTCCTCCAACCACTTGTTCAA	reverse	human	BRN2	BRN2 Exon	Detection of mRNA level by RT-qPCR	LL1735 + LL1736	Standard RT-qPCR	109
RT-Primers	LL739	ACCGTCTCTCACTGGATTGG	forward	human	MITF	M-MITF Exon 1	Detection of mRNA level by RT-qPCR	LL739 + LL740	Standard RT-qPCR	104
RT-Primers	LL740	TACTTGGTGGGGTTTTCGAG	reverse	human	MITF	M-MITF Exon 2	Detection of mRNA level by RT-qPCR	LL739 + LL740	Standard RT-qPCR	104
RT-Primers	LL1119	TCCTCCTTTTTCTTCAGCCAC	forward	human	PTEN	PTEN Exon 1	Detection of mRNA level by RT-qPCR	LL1119 + LL1120	Standard RT-qPCR	436
RT-Primers	LL1120	TCATTACACCAGTTCGTCC	reverse	human	PTEN	PTEN Exon 5	Detection of mRNA level by RT-qPCR	LL1119 + LL1120	Standard RT-qPCR	436
RT-Primers	LL152	TCTACACTGACAACTCTATCCG	forward	human	CCND1	CCND1 Exon 2	Detection of mRNA level by RT-qPCR	LL152 + LL153	Standard RT-qPCR	304
RT-Primers	LL153	TAGCAGGAGAGGAAGTTGTTGG	reverse	human	CCND1	CCND1 Exon 4	Detection of mRNA level by RT-qPCR	LL152 + LL153	Standard RT-qPCR	304
RT-Primers	LL521	CACGAACCACGGCACTGATT	forward	human	TBP	TBP Exon 5	Detection of mRNA level by RT-qPCR	LL521 + LL522	Standard RT-qPCR	
RT-Primers	LL522	TTTTCTTGCTGCCAGTCTGGAC	reverse	human	TBP	TBP Exon 6	Detection of mRNA level by RT-qPCR	LL521 + LL522	Standard RT-qPCR	
RT-Primers	LL2239	CTATGCTTACGCTTAACTCCA	forward	human	MITF	MITF Exon 2B	Detection of mRNA level by RT-qPCR	LL2239 + LL2240	Standard RT-qPCR	
RT-Primers	LL2240	TACATCATCCATCTGCATACAG	reverse	human	MITF	MITF Exon 3	Detection of mRNA level by RT-qPCR	LL2239 + LL2240	Standard RT-qPCR	
ChIP primers	LL2505	AGCGTCTAACCACTACAGCC	forward	human	BRN2	+ 11 (Exon)	BRN2 ChIP (negative control)	LL2505 + LL2506	Standard RT-qPCR	180
ChIP primers	LL2506	GGGTGTCCGTTGCTCTGC	reverse	human	BRN2	+ 161 (Exon)	BRN2 ChIP (negative control)	LL2505 + LL2506	Standard RT-qPCR	180
ChIP primers	LL1978	CAAACTCGTAGGGCTTCCAA	forward	human	M-MITF Promoter	-240 (5'-UTR)	BRN2 ChIP (positive control)	LL1978 + LL1979	Standard RT-qPCR	121
ChIP primers	LL1979	CGATACACCACCGGAAACTT	reverse	human	M-MITF Promoter	-119 (5'-UTR)	BRN2 ChIP (positive control)	LL1978 + LL1979	Standard RT-qPCR	121
ChIP primers	LL2991	CGGGTCGACTACTTGCTTTG	forward	human	PTEN Promoter	-2960 (Promoter)	BRN2 ChIP (BRN2 Binding site 1)	LL2991 + LL2992	Standard RT-qPCR	99
ChIP primers	LL2992	GTCACCGTGAGAGAGCTGAT	reverse	human	PTEN Promoter	-2861 (Promoter)	BRN2 ChIP (BRN2 Binding site 1)	LL2991 + LL2992	Standard RT-qPCR	99
ChIP primers	LL2995	GCTACCGCCAAGTCCAGA	forward	human	PTEN Promoter	-105 (5'-UTR)	BRN2 ChIP (BRN2 Binding site 2)	LL2995 + LL2996	Standard RT-qPCR	80
ChIP primers	LL2996	AAGGAGGAGAGAGATGGCAG	reverse	human	PTEN Promoter	-25 (5'-UTR)	BRN2 ChIP (BRN2 Binding site 2)	LL2995 + LL2996	Standard RT-qPCR	80
ChIP primers	LL3013	ACAGGCTCCCAGACATGAC	forward	human	PTEN Promoter	-14 (5'-UTR)	BRN2 ChIP (BRN2 Binding site 3)	LL3013 + LL3014	Standard RT-qPCR	188
ChIP primers	LL3014	AGTCACCCAAACTACGGACA	reverse	human	PTEN Promoter	+174 (1st intron)	BRN2 ChIP (BRN2 Binding site 3)	LL3013 + LL3014	Standard RT-qPCR	188
ChIP primers	LL3018	ACAGAGCGACACCCTGTCAT	forward	human	PRM1	- 1400 (5'-UTR)	M-MITF-HA ChIP (negative control)	LL3018 + LL3019	Standard RT-qPCR	104
ChIP primers	LL3019	AGGCGGTGGTTACACAACAT	reverse	human	PRM1	- 1296 (5'UTR)	M-MITF-HA ChIP (negative control)	LL3018 + LL3019	Standard RT-qPCR	104
ChIP primers	LL3016	GTGGGATACGAGCCAATTCGAAAG	forward	human	Tyrosinase Prom	- 217 (5'-UTR)	M-MITF-HA ChIP (Positive control)	LL3016 + LL3017	Standard RT-qPCR	99
ChIP primers	LL3017	TCCCACCTCCAGCATCAAACACTT	reverse	human	Tyrosinase Prom	- 118 (5'-UTR)	M-MITF-HA ChIP (Positive control)	LL3016 + LL3017	Standard RT-qPCR	99
ChIP primers	LL3020	TACACAGCCAGACCACACAT	forward	human	PTEN C-Ter region	+ 144,530	M-MITF-HA ChIP (MITF Binding site)	LL3020 + LL3021	Standard RT-qPCR	116
ChIP primers	LL3021	TGGGCCTGGACAGAATACAA	reverse	human	PTEN C-Ter region	+ 144,646	M-MITF-HA ChIP (MITF Binding site)	LL3020 + LL3021	Standard RT-qPCR	116
mouse genotyping	LL148	GAAGCAACTCATCGATTG	forward	mouse	Tyr::Cre ERt2	Cre	Genotyping	LL148 + LL622	ERT2	WT: no band, CreERt2: 140
mouse genotyping	LL622	TGAAGGGTCTGGTAGGATCA	reverse	mouse	Tyr::Cre ERt2	ERt2	Genotyping	LL148 + LL622	ERT2	WT: no band, CreERt2: 140
mouse genotyping	LL973	GCCCAGGCTCTTTATGAGAA	reverse	mouse	Braf V600E	Braf Intron 15	Genotyping	LL973 + LL996	Classic 58	WT: no band, Flox: 140
mouse genotyping	LL996	GCTTGGCTGGACGTAAACTC	forward	mouse	Braf V600E	Neomycin casette	Genotyping	LL973 + LL996	Classic 58	WT: no band, Flox: 140
mouse genotyping	LL2457	CAA GCA CTC TGC GAA CTG AG	forward	mouse	Pten	Pten Intron 4	Genotyping	LL2457 + LL2458	Classic 58	WT: 156, Flox: 328
mouse genotyping	LL2458	AAGTTTTTGAAGGCAAGATGC	reverse	mouse	Pten	Pten Intron 4	Genotyping	LL2457 + LL2458	Classic 58	WT: 156, Flox: 328
mouse genotyping	LL1927	GCGCGGCTCCTTTAACCAGAGCGCC	forward	mouse	Brn2	Brn2 5'-UTR	Genotyping	LL1927 + LL1928	Brn2	WT: 210, Flox: 250
mouse genotyping	LL1928	CTGGTGAGCGTGGCTGAGCGGGTGC	reverse	mouse	Brn2	Brn2 Exon	Genotyping	LL1927 + LL1928	Brn2	WT: 210, Flox: 250
mouse genotyping	LL972	AGTCAATCATCCACAGAGACCT	forward	mouse	Braf V600E	Braf Exon 14	Verification of Defloxing	LL972 + LL973	Classic 58	WT: 466, Flox: 518
mouse genotyping	LL973	GCCCAGGCTCTTTATGAGAA	reverse	mouse	Braf V600E	Braf Intron 15	Verification of Defloxing	LL972 + LL973	Classic 58	WT: 466, Flox: 518
mouse genotyping	LL2902	GCGGACTTGAAGAAGTCGTG	reverse	mouse	Brn2	GFP casette, 3' BRN2 exon	Verification of Defloxing	LL2902 + LL1927	Classic 58	Defloxed: 300
mouse genotyping	LL1090	ACTCAAGGCAGGGATGAGC	forward	mouse	Pten	Pten intron 4	Verification of Defloxing	LL1090 + LL1092	Classic 58	Defloxed: 300
mouse genotyping	LL1092	GCTTGATATCGAATTCCTGCAGC	reverse	mouse	Pten	Pten intron 5	Verification of Defloxing	LL1090 + LL1092	Classic 58	Defloxed: 300
RT-Primers	LL1623	AGCCCAAGGCAGAAAAGTAACT	forward	mouse	Brn2	Brn2 Exon	Detection of mRNA level by RT-qPCR	LL1623 + LL1624	Standard RT-qPCR	105
RT-Primers	LL1624	AGGCTGTAGTGGTTAGACGCTG	reverse	mouse	Brn2	Brn2 Exon	Detection of mRNA level by RT-qPCR	LL1623 + LL1624	Standard RT-qPCR	105
RT-Primers	LL833	TGAAACCTTGCTATGCTGGA	forward	mouse	Mitf	M-Mitf Exon 1	Detection of mRNA level by RT-qPCR	LL833 + LL834	Standard RT-qPCR	108
RT-Primers	LL834	TACCTGGTGCCTCTGAGCTT	reverse	mouse	Mitf	M-Mitf Exon 2	Detection of mRNA level by RT-qPCR	LL833 + LL834	Standard RT-qPCR	108
RT-Primers	LL1873	GGCGGGAGGACAAGTTCAT	forward	mouse	Pten	Pten Exon 7	Detection of mRNA level by RT-qPCR	LL1873 + LL1874	Standard RT-qPCR	110
RT-Primers	LL1874	TTTGTCCTTTTTGAGCATCTTG	reverse	mouse	Pten	Pten Exon 8	Detection of mRNA level by RT-qPCR	LL1873 + LL1874	Standard RT-qPCR	110

All location indicated relative to ATG (+1)

* see page "PCR conditions"

Supplementary Table 5. siRNA

Name	target	species	Sequences (5' -> 3')	concentration used	reference	distributor
si78	Brn2	human/ mouse	CGGAUCAAAACUGGGAUUUA<br>GCAAGGGCGCAAGCGGAAA<br>AAGGAGGUGGUGAGAGUUU<br>CGACCUUUGCAGGCGAGUAA	200 pMol	M-020029-01-0005	Dharmacon
si40	MITF	human	AGACGGAGCACACUUGUUA<br>GACCUAACCUGUACAACAA<br>GCAGAUGGAUGAUGUAAUC<br>GAACGAAGAAGAAGAUUUA	200 pMol	M-008674-00-0005	Dharmacon
si38	PTEN	human	Three siRNA to target human PTEN expression<br>Sequences undisclosed	200 pMol	sc-29459	Santa Cruz Biotech
si10	Scrambled / Control	human/ mouse	AAUUCUCCGAACGUGUCACGU-dTdT	200 pMol	Custom ordered	Eurofins Genomics

Supplementay Table 6. ChIP buffer

ChIP Buffer composition (Ci = Initial Concentration, Cf = final concentration)

**ChIP Dilution Buffer**	**Ci**	**Cf:**	**For 50mL**
SDS	20%	0.01%	25μL
Triton X-100	20%	1.1%	2.25mL
EDTA	500mM	1.2mM	120μL
Tris HCl pH 8	2M	16.7mM	417.5μL
NaCl	5M	167mM	1.67mL
H2O			45.51mL

**Low Salt Wash Buffer**	**Ci**	**Cf:**	**For 150mL**
SDS	20%	0.1%	750μL
Triton X-100	20%	1%	7.5mL
EDTA	500mM	2mM	600μL
Tris HCl pH 8	2M	20mM	1.5mL
NaCl	5M	150mM	4.5mL
H2O			135.15mL

**High Salt Wash Buffer**	**Ci**	**Cf:**	**For 150mL**
SDS	20%	0.1%	750μL
Triton X-100	20%	1%	7.5mL
EDTA	500mM	2mM	600μL
Tris HCl pH 8	2M	20mM	1.5mL
NaCl	5M	150mM	15mL
H2O			124.65mL

**LiCl Wash Buffer**	**Ci**	**Cf:**	**For 150mL**
LiCl	8M	0.25M	4.68mL
NP-40	10%	1%	15mL
EDTA	500mM	1mM	300μL
Tris HCl pH 8	2M	10mM	750μL
H2O			114.27mL

**TE Buffer**	**Ci**	**Cf:**	**For 150mL**
EDTA	500mM	1mM	300μL
Tris HCl pH 8	2M	10mM	750μL
H2O			148.95mL

**Elution buffer (Make fresh each time)**	**Ci**	**Cf:**	**For 5mL**
SDS	20%	1%	250μL
NaHCO3	0.5M	0.1M	1mL
H2O			3.75mL

**Cell Lysis buffer (can be stored at RT)**	**Ci**	**Cf:**	**For 50mL**
EDTA	500mM	10mM	1mL
Tris HCl pH 8	2M	50mM	1.25μL
SDS	20%	1%	2.5mL
H2O			45.25mL

For 5mL Cell Lysis buffer add 25μL PMSF (Sigma, P7626) and 100μL Complete Protease Inhibitor (See reference above) just before use

Supplementary Table 7. Antibodies and reagents

Antibodies (reagent / resource)	type of antibody	source	identifier
Akt	primary	Cell signaling	#4685
beta-actin	primary	Sigma	A5441
BrdU	primary	BD Biosciences	#555627
BRN2	primary	Cell signaling	#12137
CyclinD1	primary	Cell signaling	#2926
ERK (p44/42 MAP Kinase)	primary	Cell signaling	#9106
Histone H3	primary	Cell Signaling	#2650
Human influenza hemagglutinin (HA)	primary	Roche	#11583816001
Ki-67	primary	Nova-Costra	NCL-Ki67p
MITF	primary	Abcam	ab12039
normal rabbit IgG	primary	Cell Signaling	#2729
phospho plus AKT (serine 473)	primary	Cell signaling	#9271
Phospho-Akt (Ser473)	primary	Cell signaling	#3787
phospho-p42-44 MAPK (Erk1/2) (Thr202/Tyr204)	primary	Cell signaling	#4370
Phospho-S6 Ribosomal Protein (Ser235/236)	primary	Cell signaling	#4857
PTEN	primary	Cell signaling	#9559
S6	primary	Cell signaling	#2317
SOX10	primary	Abcam	ab155279
Vinculin	primary	Sigma	V9131
donkey anti-mouse Alexa 647	secondary	Abcam	ab150107
donkey anti-rabbit Alexa 488	secondary	Abcam	ab150073
donkey anti-rabbit Alexa 555	secondary	Abcam	ab150074
Goat anti-Rabbit IgG, Biotin Conjugate	secondary	Diagomics	GtxRb-003-EBIO
horseradish peroxidase-conjugated goat anti-mouse IgG	secondary	Jackson	115-035-003

horseradish peroxidase-conjugated goat anti-rabbit IgG	secondary	Jackson	111-035-003

Key resources table

Antibodies

Table key

Chemicals (REAGENT or RESOURCE)	source	identifier
3-Amino-9-ethylcarbazole (AEC)	Sigma	A6926
4′,6-diamidino-2-phenylindole (DAPI)	Sigma	D9542
Binimetinib	Selleck	S7007
bovine serum albumin	Sigma	A9418
Complete protease inhibitor	Roche	#11873580001
Hydrogenperoxide (H2O2)	Sigma	H1009
LY294002	Calbiochem	440202
Optimal cutting temperature compound (OCT)	VWR	#00411243
Paraformaldehyde (PFA)	Euromedex	15714-S
Phosphate-buffered saline (PBS)	Euromedex	ET330-A
Phostop	Roche	#04906837001
PLX4720	Axon Medchem	1474
Sodium chloride	OSI	A4321152
Sodium citrate	VWR	1120051000
sodium orthovanadate	Sigma	S6508
Tamoxifen	Sigma	T5648
Tris	Sigma	T-1503
Tween-20	VWR	8221840500

Key resources table

Chemicals

Table key

**Critical Commercial Assays (REAGENT or RESOURCE)**	**SOURCE**	**IDENTIFIER**
AllPrep DNAMini Kit	Qiagen	#80204
GeneChip 3′IVT Plus reagent Kit	Thermofisher	#902415
iTaq™ Universal SYBR Green Supermix	Bio-rad	#1725124
miRNeasy Kit	Qiagen	#217004
QIAamp DNA FFPE Tissue Kit	Qiagen	#56404
RNase-Free DNase Set	Qiagen	#79254

Key resources table

Critical Commercial Assays

Table key

**Experimental models: cell lines (REAGENT or RESOURCE)**	**SOURCE**	**IDENTIFIER**
501mel	Rambow et al., 2015	501mel
501mel HA-MITF	Strub et al., 2011	501mel HA-MITF
C57BL/6 9v melanocytes	Delmas et al., 2007	9v melanocytes
Dauv-1	Rambow et al., 2015	Dauv-1
Gerlach	Rambow et al., 2015	Gerlach
HBL	Gift from Ghanem Ghanem, Ghanem et al., 1988	HBL
Melan-a	Gift from Dorothy Bennett, Bennett et el. 1987	Melan-a
SK-Mel-28	Rambow et al., 2015	SK-Mel-28
WM793	Rambow et al., 2015	WM793

Key resources table

Experimental models cell lines

Table key

Experimental mouse models* (reagent and resource)	SOURCE	IDENTIFIER
Braf V600E/+	Dhomen et al., 2009	N/A
Brn2 flox	Jaegle et al., 2003	N/A
Pten flox	Lesche et al 2002	JAX(#006440, B6.129S4-Ptentm1Hwu/J)
Tyr::CreERT2	Yajima et al., 2006	JAX (#031281, B6N.Cg-Tg(Tyr-cre/ERT2)1Lru/J)

* all these mouse lines were backcrossed more than 10 times towards C57BL/6J

Key resources table

Experimental models organisms

Table key

REAGENT or RESOURCE	Sequence (5' -> 3')	SOURCE	IDENTIFIER
**Oligonucleotides**			
Detection of human BRN2 mRNA level by RT-qPCR (forward)	TATGGCAACGTGTTCTCGCA	This paper	LL1735
Detection of human BRN2 mRNA level by RT-qPCR (reverse)	CCTCCTCCAACCACTTGTTCAA	This paper	LL1736
Detection of human M-MITF mRNA level by RT-qPCR (forward)	ACCGTCTCTCACTGGATTGG	This paper	LL739
Detection of human M-MITF mRNA level by RT-qPCR (reverse)	TACTTGGTGGGGTTTTCGAG	This paper	LL740
Detection of human PTEN mRNA level by RT-qPCR (forward)	TCCTCCTTTTTCTTCAGCCAC	This paper	LL1119
Detection of human PTEN mRNA level by RT-qPCR (reverse)	TCATTACACCAGTTCGTCC	This paper	LL1120
Detection of human CyclinD1 mRNA level by RT-qPCR (forward)	TCTACACTGACAACTCTATCCG	Dziadek and Adamson (1978) JEEM 8, 152-160	LL152
Detection of human CyclinD1 mRNA level by RT-qPCR (reverse)	TAGCAGGAGAGGAAGTTGTTGG	Dziadek and Adamson (1978) JEEM 8, 152-160	LL153
Detection of human TBP mRNA level by RT-qPCR (forward)	CACGAACCACGGCACTGATT	This paper	LL521
Detection of human TBP mRNA level by RT-qPCR (reverse)	TTTTCTTGCTGCCAGTCTGGAC	This paper	LL522
Detection of human MITF mRNA level by RT-qPCR (forward)	CTATGCTTACGCTTAACTCCA	This paper	LL2239
Detection of human MITF mRNA level by RT-qPCR (reverse)	TACATCATCCATCTGCATACAG	This paper	LL2240
BRN2 ChIP (BRN2 CDS, negative control) (forward)	AGCGTCTAACCACTACAGCC	This paper	LL2505
BRN2 ChIP (BRN2 CDS, negative control) (reverse)	GGGTGTCCGTTGCTCTGC	This paper	LL2506
BRN2 ChIP (MITF promoter, positive control) (forward)	CAAACTCGTAGGGCTTCCAA	Berlin et al. 2012	LL1978
BRN2 ChIP (MITF promoter, positive control) (reverse)	CGATACACCACCGGAAACTT	Berlin et al. 2012	LL1979
BRN2 ChIP (BRN2 Binding site 1) (forward)	CGGGTCGACTACTTGCTTTG	This paper	LL2991
BRN2 ChIP (BRN2 Binding site 1) (reverse)	GTCACCGTGAGAGAGCTGAT	This paper	LL2992
BRN2 ChIP (BRN2 Binding site 2) (forward)	GCTACCGCCAAGTCCAGA	This paper	LL2995
BRN2 ChIP (BRN2 Binding site 2) (reverse)	AAGGAGGAGAGAGATGGCAG	This paper	LL2996
M-MITF-HA ChIP (PRM1, negative control) (forward)	ACAGAGCGACACCCTGTCAT	Laurette et al. 2015	LL3018
M-MITF-HA ChIP (PRM1, negative control) (reverse)	AGGCGGTGGTTACACAACAT	Laurette et al. 2015	LL3019
M-MITF-HA ChIP (Tyrosinase promoter, positive control) (forward)	GTGGGATACGAGCCAATTCGAAAG	Mehdi et al. 2010	LL3016
M-MITF-HA ChIP (Tyrosinase promoter, positive control) (reverse)	TCCCACCTCCAGCATCAAACACTT	Mehdi et al. 2010	LL3017
M-MITF-HA ChIP (MITF Binding site) (forward)	TACACAGCCAGACCACACAT	This paper	LL3020
M-MITF-HA ChIP (MITF Binding site) (reverse)	TGGGCCTGGACAGAATACAA	This paper	LL3021
Genotyping Tyr::Cre ERt2 mice (forward)	GAAGCAACTCATCGATTG	Dhomen et al. 2009	LL148
Genotyping Tyr::Cre ERt2 mice (reverse)	TGAAGGGTCTGGTAGGATCA	Dhomen et al. 2009	LL622
Genotyping BrafV600E mice (forward)	GCTTGGCTGGACGTAAACTC	Dhomen et al. 2009	LL996
Genotyping BrafV600E mice (reverse)	GCCCAGGCTCTTTATGAGAA	Dhomen et al. 2009	LL973
Genotyping Pten flox mice (forward)	CAA GCA CTC TGC GAA CTG AG	This paper	LL2457
Genotyping Pten flox mice (reverse)	AAG TTT TTG AAG GCA AGA TGC	This paper	LL2458
Genotyping Brn2 flox mice (forward)	GCGCGGCTCCTTTAACCAGAGCGCC	Jaegle et al. 2003	LL1927
Genotyping Brn2 flox mice (reverse)	CTGGTGAGCGTGGCTGAGCGGGTGC	Jaegle et al. 2003	LL1928

Verification of Braf Defloxing (forward)	AGTCAATCATCCACAGAGACCT	Dhomen et al. 2009	LL972
Verification of Braf Defloxing (reverse)	GCCCAGGCTCTTTATGAGAA	Dhomen et al. 2009	LL973
Verification of Brn2 Defloxing (reverse)	GCGGACTTGAAGAAGTCGTG	This paper	LL2902
Verification of Pten Defloxing (forward)	ACTCAAGGCAGGGATGAGC	Groszer et al. 2001	LL1090
Verification of Pten Defloxing (reverse)	GCTTGATATCGAATTCCTGCAGC	Groszer et al. 2001	LL1092
Verification of Brn2 Defloxing (reverse)	GAACTTCAGGGTCAGCTTGCCGTAGG	Jaegle et al. 2003	LL1971
Detection of mouse Brn2 mRNA level by RT-qPCR (forward)	AGCCCAAGGCAGAAAAGTAACT	Berlin et al. 2012	LL1623
Detection of mouse Brn2 mRNA level by RT-qPCR (reverse)	AGGCTGTAGTGGTTAGACGCTG	Berlin et al. 2012	LL1624
Detection of mouse Mitf mRNA level by RT-qPCR (forward)	TGAAACCTTGCTATGCTGGA	This paper	LL833
Detection of mouse Mitf mRNA level by RT-qPCR (reverse)	TACCTGGTGCCTCTGAGCTT	This paper	LL834
Detection of mouse Pten mRNA level by RT-qPCR (forward)	GGCGGGAGGACAAGTTCAT	This paper	LL1873
Detection of mouse Pten mRNA level by RT-qPCR (reverse)	TTTGTCCTTTTTGAGCATCTTG	This paper	LL1874

Key resources table
Oligonucleotides

Table key

REAGENT or RESOURCE

Software and Algorithms

ZEISS ZEN 2.5 V1.0
Adobe Photoshop CS6
Adobe Illustrator CS6
ImageJ 1.47v
GraphPad PRISM V6.0
edgeR
Plier
Gene Set Enrichment Analysis
cbioportal

Key resources table

Software and Algorithms

SOURCE

Carl Zeiss AG
Adobe Systems
Adobe Systems
Public domain
GraphPad Software, Inc
Robinson et al. *Bioinformatics* (2010) **26**(1), 139-140.
Inc. A, Miller CJ, PICR (2018). *plier: Implements the Affymetrix PLIER algorithm*. R package version 1.50.0.
Broad Institute
Gao et al. Sci. Signal. 2013 & Cerami et al. Cancer Discov. 2012

IDENTIFIER

https://www.zeiss.com/microscopy/int/products/microscope-software/zen.html

https://imagej.nih.gov/ij/

http://www.bioconductor.org
http://www.bioconductor.org
http://software.broadinstitute.org/gsea/msigdb/index.jsp
http://www.cbioportal.org/
