# Supplementary material for: BRN2 is a non-canonical melanoma tumor-suppressor
Source: Nat Commun. 2021 Jun 17;12:3707. doi: 10.1038/s41467-021-23973-5 (PMC8211827; doi:10.1038/s41467-021-23973-5)
Supplement: Supplementary file 3 — Description of Additional Supplementary Files [file 41467_2021_23973_MOESM3_ESM.pdf]

Description of Additional Supplementary Files

File Name: Supplementary Data 1
Description: aCGH (Comparative Genomic Hybridization) of 23 human melanoma cell lines. Statistical test was performed using the two-tailed unpaired t-test from three independent cell passages.

File Name: Supplementary Data 2
Description: Genomic mono anti-allelic deletion of a series of human melanoma (TCGA) in 6q region.

File Name: Supplementary Data 3
Description: Microarray of Braf-Pten-Brn2-WT/het/hom mouse melanoma tumors.
The two-sided differential expression analysis was conducted using limma – a linear model followed by an empirical Bayesian analysis. The p-value were then adjusted using the Benjamini-Hochberg (FDR).

File Name: Supplementary Data 4
Description: Microarray of Braf-Pten-Brn2-WT/het/hom mouse melanoma cell lines.
The two-sided differential expression analysis was conducted using limma – a linear model followed by an empirical Bayesian analysis. The p-value were then adjusted using the Benjamini-Hochberg (FDR).
