# Supplementary material for: BRN2 is a non-canonical melanoma tumor-suppressor
Source: Nat Commun. 2021 Jun 17;12:3707. doi: 10.1038/s41467-021-23973-5 (PMC8211827; doi:10.1038/s41467-021-23973-5)
Supplement: Supplementary file 8 — Reporting Summary [file 41467_2021_23973_MOESM8_ESM.pdf]

nature research

Corresponding author(s): Lionel LARUE,

Last updated by author(s): Apr 28, 2021

Reporting Summary

Nature Research wishes to improve the reproducibility of the work that we publish. This form provides structure for consistency and transparency in reporting. For further information on Nature Research policies, see our Editorial Policies and the Editorial Policy Checklist.

Statistics

For all statistical analyses, confirm that the following items are present in the figure legend, table legend, main text, or Methods section.

n/a	Confirmed	
☐	☒	The exact sample size (*n*) for each experimental group/condition, given as a discrete number and unit of measurement
☐	☒	A statement on whether measurements were taken from distinct samples or whether the same sample was measured repeatedly
☐	☒	The statistical test(s) used AND whether they are one- or two-sided<br>*Only common tests should be described solely by name; describe more complex techniques in the Methods section.*
☒	☐	A description of all covariates tested
☒	☐	A description of any assumptions or corrections, such as tests of normality and adjustment for multiple comparisons
☐	☒	A full description of the statistical parameters including central tendency (e.g. means) or other basic estimates (e.g. regression coefficient) AND variation (e.g. standard deviation) or associated estimates of uncertainty (e.g. confidence intervals)
☐	☒	For null hypothesis testing, the test statistic (e.g. *F*, *t*, *r*) with confidence intervals, effect sizes, degrees of freedom and *P* value noted<br>*Give P values as exact values whenever suitable.*
☒	☐	For Bayesian analysis, information on the choice of priors and Markov chain Monte Carlo settings
☒	☐	For hierarchical and complex designs, identification of the appropriate level for tests and full reporting of outcomes
☒	☐	Estimates of effect sizes (e.g. Cohen's *d*, Pearson's *r*), indicating how they were calculated

Our web collection on statistics for biologists contains articles on many of the points above.

Software and code

Policy information about availability of computer code

Data collection	Zeiss ZEN 2.5 V1.0, cbioportal, Microsoft Power point, Doc , xls
Data analysis	Adobe Photoshop CS6, Adobe Illustrator CS6, Image J 1.47v, GraphPad Prism 6.0, edgeR, Plier, Gene Set Enrichment Analysis, xls

For manuscripts utilizing custom algorithms or software that are central to the research but not yet described in published literature, software must be made available to editors and reviewers. We strongly encourage code deposition in a community repository (e.g. GitHub). See the Nature Research guidelines for submitting code & software for further information.

Data

Policy information about availability of data

All manuscripts must include a data availability statement. This statement should provide the following information, where applicable:

- Accession codes, unique identifiers, or web links for publicly available datasets
- A list of figures that have associated raw data
- A description of any restrictions on data availability

- Accession codes are given in the text : GSE163086, GSE163085, GSE126524, are associated with figure 4 and figure S5, and GSE153020 is associated with figure 6.
- Figures S8 and S9 are associated with raw data.

Field-specific reporting

Please select the one below that is the best fit for your research. If you are not sure, read the appropriate sections before making your selection.

☒ Life sciences ☐ Behavioural & social sciences ☐ Ecological, evolutionary & environmental sciences

For a reference copy of the document with all sections, see nature.com/documents/nr-reporting-summary-flat.pdf

Life sciences study design

All studies must disclose on these points even when the disclosure is negative.

Sample size	The number of mice of a specific genotype was equal or superior to 6. This number is based on our experience on a pur C57BL/6 background.
Data exclusions	No data was excluded from the analyses
Replication	Each experiment was performed independently at least three times. Thay always went to the same direction.
Randomization	Each mutation was maintained by backcrossing the mice on C57BL/6J. Mice were generated and genotyped after multiple crosses. Littermates were either induced with tamoxifen or not in a random way on days 1-3. Genotypes of the animals were unknown at that stage. Treatments of cell cultures were randomly performed genetically or pharmacologically.
Blinding	See above for mouse experiments. For instance the number of Ki-67 and BrdU positive cells (see Figure 3) were done blindly by independent experimentators.

Reporting for specific materials, systems and methods

We require information from authors about some types of materials, experimental systems and methods used in many studies. Here, indicate whether each material, system or method listed is relevant to your study. If you are not sure if a list item applies to your research, read the appropriate section before selecting a response.

Materials & experimental systems

n/a	Involved in the study	
☐	☒	Antibodies
☐	☒	Eukaryotic cell lines
☒	☐	Palaeontology and archaeology
☐	☒	Animals and other organisms
☒	☐	Human research participants
☒	☐	Clinical data
☒	☐	Dual use research of concern

Methods

n/a	Involved in the study	
☐	☒	ChIP-seq
☒	☐	Flow cytometry
☒	☐	MRI-based neuroimaging

Antibodies

Antibodies used	See 1 Table key 2020 1207
Validation	We used 17 commercial primary antibodies that were validated by the companies for the appropriate experiments. It includes Cell Signaling, Sigman, BD Biosciences, Roche, Nova-Costra, Abcam. The entire information can be found in Table key that is available as supplemental information. Of course, we performed appropriate negative and positive controls for each antibody.

Eukaryotic cell lines

Policy information about cell lines

Cell line source(s)	See 1 Table key 2020 1207
Authentication	Transcriptomic analysis of all human and mouse cell lines were performed and available
Mycoplasma contamination	All cell lines are routinely tested and are negative for mycoplasma. (MycoAlert - Lonza)
Commonly misidentified lines (See ICLAC register)	None

Animals and other organisms

Policy information about studies involving animals; ARRIVE guidelines recommended for reporting animal research

Laboratory animals	Mouse - C57BL/6J males and females - transgenic animals. Mice were kept up to 18 months. The temperature is around 22°C with 60% humidity.
Wild animals	The study did not involve wild animals
Field-collected samples	No field-collected samples were used in this study
Ethics oversight	Mice were bred and maintained in the specific pathogen-free mouse colony of the Institut Curie, in accordance with the institute's regulations and French and European Union laws. Experimental procedures were specifically approved by the ethics committee of the Institut Curie CEEA-IC #118 (CEEA-IC 2016-001) in compliance with the international guidelines.

Note that full information on the approval of the study protocol must also be provided in the manuscript.

ChIP-seq

Data deposition

☒ Confirm that both raw and final processed data have been deposited in a public database such as GEO.

☒ Confirm that you have deposited or provided access to graph files (e.g. BED files) for the called peaks.

Data access links<br>*May remain private before publication.*	https://www.ncbi.nlm.nih.gov/geo/query/acc.cgi?acc=GSE153020<br>Enter token yjcxoogixbwljuj into the box
Files in database submission	GSM4633173 WT_MITF_CnR_rep1<br>GSM4633174 WT_MITF_CnR_rep2<br>GSM4633179 MITF_mutant_MITF_CnR_rep1<br>GSM4633180 MITF_mutant_MITF_CnR_rep2<br>GSM4633181 WT_IgG_CnR_rep1<br>GSM4633182 WT_IgG_CnR_rep2<br>GSM4633187 MITF_mutant_IgG_CnR_rep1<br>GSM4633188 MITF_mutant_IgG_CnR_rep2
Genome browser session<br>(e.g. UCSC)	https://genome.ucsc.edu/s/cpknny/MITF_CnR_PTEN

Methodology

Replicates

Each CUT&RUN experiment was preformed in duplicate. MITF was targeted in wild type and MITF mutant cell lines. MITF peaks were identified as those present in wild type cells and absence from mutant cells. IgG was used as a background control.

Sequencing depth

CUT&RUN requires very low sequence read depth (5-10 million reads per sample), we sequenced all samples to an average of 15 million paired reads per sample. Paired end 2x150bp reads were sequenced on the HiSeq X platform.
BAM mapping statistics are as follow:
Summary statistics on proceeded BAM files below, BAM files were filtered to capture transcription factor binding sites (fragment length for valid paired-end alignment 120bp), as described by Skene and Henikoff 2017 elife.

CUT&RUN_MITF_wild type_rep 1:
Total records: 11107186: QC failed: 0, Optical/PCR duplicate: 0, Non primary hits 0, Unmapped reads: 0, mapq < mapq_cut (non-unique): 2630778, mapq >= mapq_cut (unique)8476408, Read-1: 4238204, Read-2: 4238204,Reads map to '+': 4238204,Reads map to '-': 4238204, Non-splice reads: 8476408, Splice reads:0, Reads mapped in proper pairs: 8476408, Proper-paired reads map to different chrom:0.

CUT&RUN_MITF_wild type_rep 2:
Total records: 9979866,QC failed: 0, Optical/PCR duplicate: 0, Non primary hits 0, Unmapped reads: 0, mapq < mapq_cut (non-unique): 2342700, mapq >= mapq_cut (unique): 7637166, Read-1: 3818583, Read-2: 3818583, Reads map to '+': 3818583, Reads map to '-': 3818583, Non-splice reads: 7637166, Splice reads: 0, Reads mapped in proper pairs: 7637166, Proper-paired reads map to different chrom: 0

CUT&RUN_MITF_mutant_rep 1:
Total records: 24030236, QC failed: 0, Optical/PCR duplicate: 0, Non primary hits0, Unmapped reads: 0, mapq < mapq_cut (non-unique): 4924524, mapq >= mapq_cut (unique): 19105712,Read-1: 9552856, Read-2: 9552856, Reads map to '+': 9552856, Reads map to '-': 9552856, Non-splice reads: 19105712, Splice reads: 0, Reads mapped in proper pairs: 19105712, Proper-paired reads map to different chrom:0

CUT&RUN_MITF_mutant_rep 2:
Total records: 24325554, QC failed: 0, Optical/PCR duplicate: 0, Non primary hits0, Unmapped reads: 0, mapq < mapq_cut (non-unique): 4902720, mapq >= mapq_cut (unique): 19422834, Read-1: 9711417, Read-2: 9711417, Reads map to '+': 9711417, Reads map to '-': 9711417, Non-splice reads: 19422834, Splice reads: 0, Reads mapped in, proper pairs: 19422834, Proper-paired reads

map to different chrom:0

Antibodies	MITF: HPA003259-100ul Anti MITF, Lot B57783, 0.1mg/ml, Sigma<br>IgG: Normal Rabbit IgG EMD Millipore 12-370, Lot 3202364
Peak calling parameters	MACS2 "callpeak" in bedtools was used for peak detection. Tool Version macs2 2.1.1.20160309. CUT&RUN samples were used as input and IgG for background control. Parameters: Format of Input Files - Paired-end BAM, Set lower mfold bound 5, Set upper mfold bound 50. Band width for picking regions to compute fragment size 150, Peak detection based on qvalue, Minimum FDR (q-value) cutoff for peak detection 0.05. Advanced _options - Default. Peaks detected in wild type samples and not in MITF-knockouts were considered true-MITF peaks. BED files will be uploaded to GSE153020
Data quality	Peaks with FDR < 0.05, lower mfold >5 and upper mfold <50 were only considered in this analysis.
Software	All data was processed on Galaxy - usegalaxy.org.<br>Software used;<br>FastQC Read Quality reports (Galaxy Version 0.72+galaxy1)<br>Trim Galore! Quality and adapter trimmer of reads (Galaxy Version 0.6.3)<br>Bowtie2 - map reads against reference genome (Galaxy Version 2.3.4.3+galaxy0)<br>BAM filter Removes reads from a BAM file based on criteria (Galaxy Version 0.5.9)<br>MACS2 callpeak Call peaks from alignment results (Galaxy Version 2.1.1.20160309.6)<br>Differential peak analysis<br>DiffBind differential binding analysis of ChIP-Seq peak data (Galaxy Version 2.10.0<br>Data visualization,<br>Deeptools, multiBigwigSummary, bamCoverage on command line.
